# Physiology and molecular biology of aquatic cyanobacteria

**DOI:** 10.3389/fmicb.2014.00359

**Published:** 2014-07-16

**Authors:** George S. Bullerjahn, Anton F. Post

**Affiliations:** ^1^Department of Biological Sciences, Bowling Green State UniversityBowling Green, OH, USA; ^2^Marine Biological Laboratory Woods HoleWoods Hole, MA, USA

**Keywords:** cyanobacteria, HABs, Prochlorococcus, Synechococcus, nitrogen fixation

Cyanobacteria thrive in every illuminated aquatic environment known, contributing at least 25% of primary productivity worldwide. Given their importance in carbon and nutrient cycles, cyanobacteria are essential geochemical agents that have shaped the composition of the Earth's crust, oceans and atmosphere for billions of years. The high diversity of cyanobacteria is reflected in the panoply of unique physiological adaptations across the phylum, including different strategies to optimize light harvesting or sustain nitrogen fixation, but also different lifestyles like psychrotrophy, and oligotrophy. Some cyanobacteria produce secondary metabolites of cryptic function, many of which are toxic to eukaryotes. Consequently, bloom-forming toxic cyanobacteria are global hazards that are of increasing concern in surface waters affected by anthropogenic nutrient loads and climate change. While focusing on cyanobacteria in aquatic environments, the collection of papers herein touches on this broad range of topics.

Regarding the role of cyanobacteria in the ocean, the importance of the unicellular picocyanobacteria is paramount. Indeed, *Synechococcus* and *Prochlorococcus* are the abundant genera in the oligotrophic open sea. Given that cyanobacteria are under regular assault imposed by photooxidative stresses, and that such stresses constrain photosynthetic performance, the paper by Mella-Flores et al. (2012) describes different strategies employed by model strains of *Synechococcus* and *Prochlorococcus* to high light and UV. Nonetheless, in the ocean, *Synechococcus* is composed of several clades that likely define particular ecotypes. Detection and enumeration of distinct *Synechococcus* clades is essential in understanding how these organisms co-exist with one another. To this end, Ahlgren and Rocap (2012) developed a suite of qPCR tools enabling a thorough analysis of *Synechococcus* community structure and dynamics.

Nitrogen fixation by the unicellular *Crocosphaera watsonii* is now recognized to be a significant source of new N to marine environments. Two papers describe physiological properties of *Crocosphaera* relevant to their ecological importance. Sohm et al. (2011) demonstrate that a strain of *C. watsonii* produces high amounts of extracellular polysaccharides. The high level of organic carbon production suggests that this strain may directly support significant heterotrophy in the marine C cycle. Given that arsenate is a toxic anion that can interfere with the transport and metabolism of phosphate, Dyhrman and Haley (2011) report mechanisms of arsenate resistance in *Crocosphaera*, whose habitat includes P-limited ocean gyres. Such regions often have high arsenate:phosphate ratios, thus detoxification mechanisms are an important strategy for N fixation in these oligotrophic regions.

The structure and physiological performance of arctic cyanobacterial mats is of particular interest, given their dominant contribution to biomass at high latitudes, and the stresses imposed by global warming on such fragile environments. Lionard et al. (2012) examine the composition of an artic microbial mat, and demonstrate the robust adaptation of the cyanobacteria to osmotic stress, a likely outcome of climate change.

The characterization of chlorophyll-*b* containing *Prochlorothrix* spp. by Pinevich et al. (2012) indicate that despite the scarcity of this genus in aquatic environments, molecular methods suggest it is more widely distributed. The authors compare the physiological characteristics of the two known species and discuss the evolution of their light harvesting apparatus.

Four papers explore the physiology of bloom-forming and toxic cyanobacteria. Kaplan et al. (2012) address the enigmatic function of the microcystins and cylindrospermopsins, describing experimental evidence for their role in cell-cell communication among bloom-formers. Sukenik et al. (2012) explore the spread of *Aphanizomenon* and *Cylindrospermopsis* to temperate freshwater environments. The appearance of such blooms is a likely consequence of global warming along with anthropogenic nutrient load, leading to a sustained invasion of into the middle latitudes. Given that management of phosphorus inputs is a strategy for limiting algal blooms in freshwaters, Saxton et al. (2012) describe the phosphorus quotas (intracellular and total) of the toxic colonial cyanobacterium, *Microcystis aeruginosa*. In response to increased phosphorus, *Microcystis* exhibited a higher cell P quota, yet a stable growth rate compared to lower phosphorus treatments. Such work has implications in modeling *Microcystis* blooms from P loadings into freshwaters. Gagnon and Pick (2012) contribute a study showing that the potent neurotoxin anatoxin-a is associated with nitrogen availability in *Aphanizomenon*. Understanding environmental cues for toxin production is essential in ultimately mitigating bloom toxigenicity.

Together, these important contributions cover many of the issues tackled in the field today: the role of cyanobacteria in the global carbon and nutrient cycles, cyanobacterial evolution, the adaptation of cyanobacteria to climate change and the consequences of toxigenicity. The studies described in this collection provide an excellent introduction to these topics, while providing important clues regarding the future directions that studies of cyanobacteria will take.

## Conflict of interest statement

The authors declare that the research was conducted in the absence of any commercial or financial relationships that could be construed as a potential conflict of interest.
